# Venous Gas Embolism During Radical Robotic Prostatectomy: A Case Report and Evidence-Based Management Algorithm

**DOI:** 10.7759/cureus.17296

**Published:** 2021-08-19

**Authors:** Courtney Vidovich, Andres Laserna, Suzanne B Karan

**Affiliations:** 1 Anesthesiology and Perioperative Medicine, University of Rochester School of Medicine and Dentistry, Rochester, USA; 2 Anesthesiology, University of Rochester, Rochester, USA

**Keywords:** venous air embolism, robotic prostatectomy, general anesthesia, iatrogenic complication, adverse event

## Abstract

Robotic-assisted radical prostatectomy (RARP) has gained rapid popularity in the last two decades after early reports of excellent survival rates, quick learning curves, and minimal invasion or tissue damage. Given the anatomical location of surgical prostatectomies and the utilization of intra-abdominal gas during laparoscopy, there is a risk of developing venous air embolism (VAE). We present a case of a 62-year-old male with hypothyroidism and benign prostatic hyperplasia who underwent robotic suprapubic prostatectomy under general anesthesia. One hour after incision the ETCO_2_ suddenly dropped (40 mmHg to 25 mmHg) as did the SpO2 (98% to 90%). There were no other vital sign changes, nor was there significant blood loss. The surgical team was notified, which prompted the surgeon to inform us that he had just been dissecting around the pelvic venous plexus. At this point, with the clinical suspicion of VAE, abdominal insufflation pressure was lowered, and inspired oxygen was increased to 100%. After 10 minutes, SpO2 and ETCO2 normalized. A debrief and literature review inspired us to develop a laparoscopic-specific VAE management algorithm, with attention to robotic-case management issues. To the best of our knowledge, this is a rare case report describing a clinical VAE during RARP.

## Introduction

Robotic-assisted radical prostatectomy (RARP) has gained popularity in the last two decades after early reports of excellent survival rates, quick learning curves, and minimal invasion or tissue damage [1]. Some argue that RARP is the new gold standard for the management of localized prostate cancer citing advantages over open radical retropubic prostatectomy (RRP) and laparoscopic retropubic prostatectomy (LRP) including decreased transfusions and conversions to open surgery, shorter hospital stay, improved one-year continence, and decreased complications when compared specifically to RRP outcomes [2,3].

Given the anatomical location of surgical prostatectomies and the utilization of intra-abdominal gas during laparoscopy, there is a risk of developing venous air embolism (VAE). While there have been a number of recent cases reporting clinical VAE using a variety of prostatectomy techniques including RRP [4], transurethral resection of prostate (TURP) [5], holmium laser enucleation of the prostate (HoLEP) [6], and greenlight laser photovaporizer (GLPV) [7], to the best of our knowledge, this is a rare case report describing a clinical VAE during RARP.

Appropriate and efficacious management of VAE has previously been outlined [8] and revised [9]. Although these reviews are thorough and provide a comprehensive foundation for VAE prevention and management, there is a need for procedure-specific management algorithms as a reference to provide efficient, procedure-tailored, and standardized patient care. After the description of the case report, we present a literature review of VAE management during laparoscopic surgeries and an evidence-based algorithm for the management of VAE during robotic laparoscopies. The patient in our case provided consent for publication of this report.

## Case presentation

**Figure 1:**
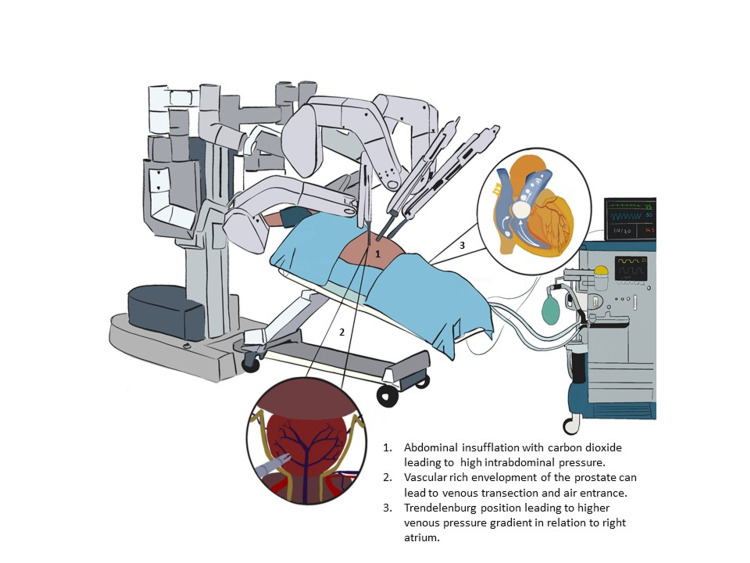
Schematic of RARP VAE evolution RARP, Robotic-assisted radical prostatectomy; VAE, venous air embolism.

A 62-year-old male with a history significant for medication-controlled hypothyroidism and benign prostatic hyperplasia presented for robotic suprapubic prostatectomy under general anesthesia; the schematic is shown in Figure 1. After uneventful induction and intubation, an arterial line was placed for pulse-pressure-variation (PPV)-targeted fluid administration. The patient was positioned in steep Trendelenburg. One hour after incision, during transection of the pelvic venous plexus, the patient’s end-tidal carbon dioxide (ETCO_2_) dropped from 40 mmHg to 25 mmHg in a single breath, and oxygen saturation (SpO_2_) decreased from 98% to 90%. There were no changes in the electrocardiogram, blood pressure, or ventilation, nor was there any concomitant blood loss. The surgical team was notified and decreased abdominal insufflation pressures while the patient’s inspired oxygen was increased to 100%. After 10 minutes, SpO_2_ and ETCO_2_ normalized. As this presentation was highly suspicious for CO_2_ venous embolism, the emergence plan was modified to exclude the use of nitrous oxide. After tracheal extubation, the patient was transferred to the post-anesthesia care unit where he recovered successfully and was discharged home on the same day.

## Discussion

We present the successful management of VAE during robotic-assisted prostatectomy characterized by timely identification, proper communication, and adequate support, resulting in no further decompensation. After debriefing and reflecting on the case, we conducted a literature review using Medline and Embase between January 2010 and July 2020. No restrictions on language or publication date were employed, and the terms used were "Prostatectomy," "Robotic prostatectomy," “Laparoscopy,” combined with “Air Embolism,” “Embolism," “CO_2_ embolism," excluding the word "embolization." Our inclusion criteria were articles in which VAE was considered or discussed. Two authors (AL and CV) independently screened citations from the initial search using a two-step approach in which first the title and then the abstracts were screened for eligibility using the software Abstrackr (Brown University, School of Public Health, Providence, USA) [10]. For citations that were considered potentially relevant, the full text was retrieved and further screened for eligibility. In cases of disagreement, both reviewers discussed and achieved consensus, and consulted with the third author (SK) to include or exclude articles. After a full-text review and data extraction, we developed a management algorithm for VAE during robotic laparoscopies.

**Figure 2:**
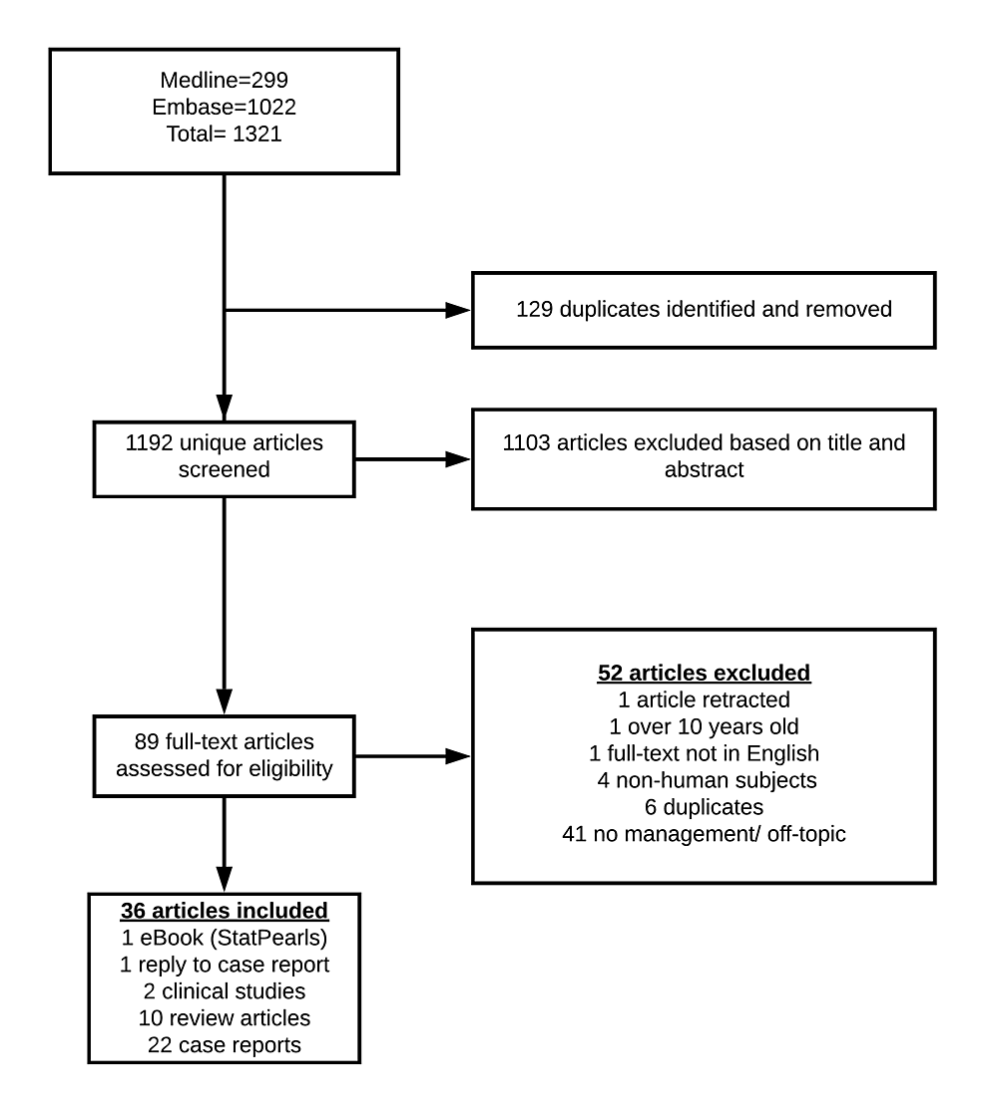
Literature review of VAE and RARP case management RARP, Robotic-assisted radical prostatectomy; VAE, venous air embolism.

We extracted 37 articles largely comprised of review articles and case reports (Figure 2).

These articles originate from around the world and include 11 from the United States; five from Japan; four from Korea; three each from Australia and Italy; two each from China, India, and Turkey; and one each from Denmark, Singapore, Spain, the Netherlands, and the United Kingdom. All 37 articles consider VAE management during laparoscopic procedures and include four robotic laparoscopies.

Risk of VAE and pathophysiology

We did not find any published reports of VAE occurring during RARP. Although the risk of a clinically significant VAE is considered rare, it has been a well-established risk of laparoscopic surgeries with a high reported mortality rate of 28% [8,9,11]. Subclinical VAEs diagnosed with transesophageal echocardiogram (TEE) have been reported at an incidence of 17%-38% [12]. This risk of VAE and its effect on morbidity and mortality are dependent on the rate and volume of air accumulation, which varies by degree of vasculature exposed and the pressure gradient between the exposed venous system and the right heart [8,9,13-15].

The degree of vasculature exposed to air varies by procedure. For prostatectomies, the vascular-rich envelopment of the prostate increases the level of risk even with the minimally invasive RARP approach [16] with one study reporting 100% of subclinical embolic events during RARP occurring during transection of the deep dorsal venous complex [12]. The most commonly reported risk factor, gas insufflation, was mentioned in 73% of the case reports reviewed [6,7,9,17-30]. The schematic in Figure 1 highlights several of these risks.

Intraoperative diagnosis

While the application of the American Society of Anesthesia-guided monitoring is the standard for such procedures [9,17,18], it has been noted to be equally essential that this monitoring be paired with a vigilant anesthesiologist [8,9,19,20].

The diagnosis of VAE in anesthetized patients mostly presents as tachyarrhythmia, right heart strain, hypotension, decreased end-tidal carbon dioxide and oxygen saturation, and, in severe cases, neurologic sequelae that in most cases are masked until the postoperative period [8,9]. Furthermore, the presentation of VAE varies by size and rate, making its detection challenging, though adverse signs and symptoms have been elucidated based on the size of air entrainment [8,17].

Though TEE is a highly sensitive technique used for the diagnosis of VAE with a detection capability of 0.02 ml/kg of entrained air, it is invasive, expensive, and requires expertise [8,21]. Pulmonary artery catheterization has been used to detect air emboli of 0.25 ml/kg, although placement without other indications may cause more risk than warranted [8]. The use of a precordial Doppler ultrasound is highly sensitive in detecting emboli as small as 0.05 ml/kg, most cost-effective, easy to use, and least invasive [8,22]. Auscultation of a "mill-wheel" murmurs over the precordium rules in VAE [18]. Even with these options, these technologies used as a tool for VAE detection were referenced in 47% of case reports [9,14,23-31].

Management: communication and robotic considerations

Communication with the perioperative team improves the timely anticipation of high-risk VAE portions of surgery [8]. If VAE is suspected by the anesthesiologist, it is imperative to inform the surgeon and start maneuvers such as flooding the exposed vasculature with saline and decreasing insufflation pressure. While flooding the surgical field is effective, it was not mentioned as a common practice in the laparoscopic case reports [9,19,28,32,33].

Decreasing insufflation pressures is more commonly described, likely due to ease of implementation [6,7,9,20,26,28-30,32-39]. While these maneuvers are both effective, hemodynamic support is simultaneously managed by the anesthesiologist with concomitant administration of intravenous fluids, vasopressors, and 100% oxygen to maximize the patient’s respiratory mismatch [6,7,9,17,18,20,23-25,28,29,32,33,36-38,40-46]. Avoidance of nitrous oxide was more likely to be recommended if patient risk factors were already present, like a patent foramen ovale, as a way to lessen the likelihood of increasing the size of entrained air [8,9,40].

Maneuvers that encourage the release of the air from the right ventricular outflow tract are debatable [19] but still recommended [8,17,41]. These maneuvers, most notably the Trendelenburg or Durant maneuver (Trendelenburg plus partial left lateral decubitus position), were reported in 69% of case reports reviewed and were considered in most review articles [9,14,17,18,20,23,24,28,29,33,35,37-39,42,43].

**Figure 3:**
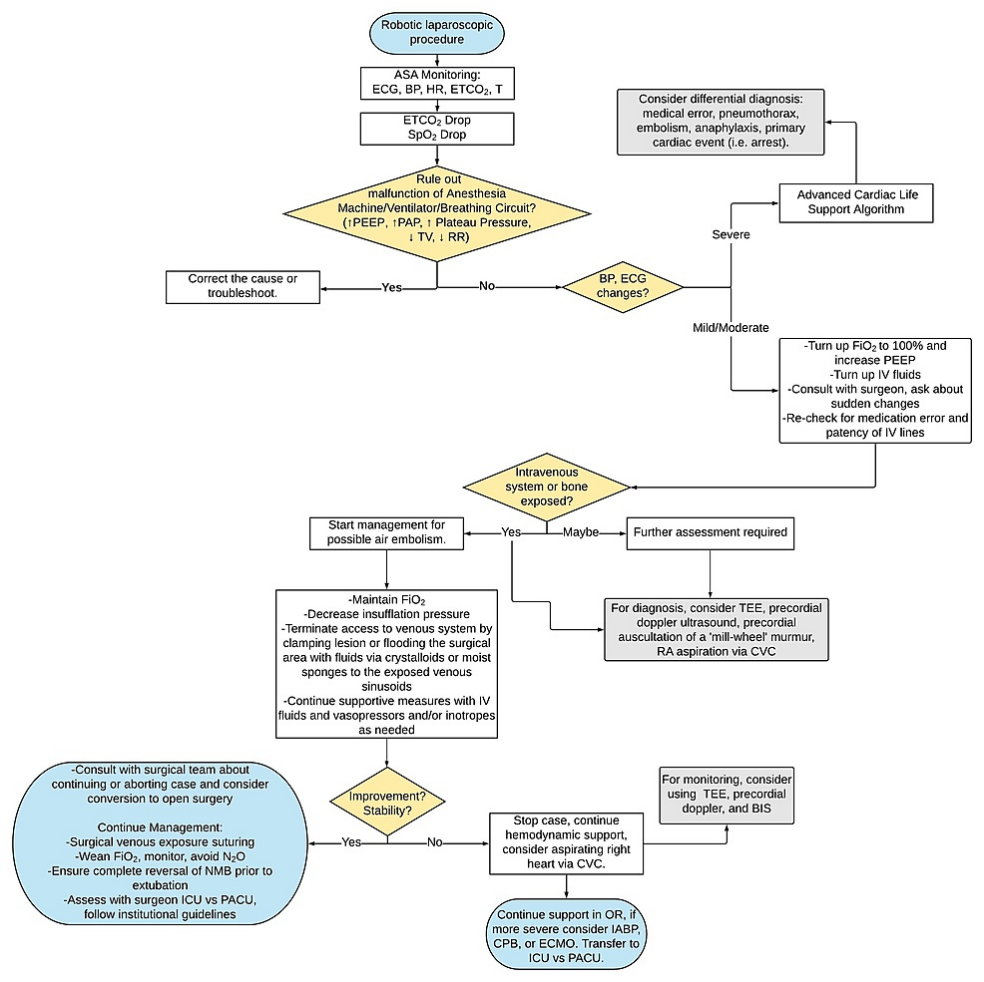
Management algorithm for VAE during RARP RARP, Robotic-assisted radical prostatectomy; VAE, venous air embolism.

The use of Trendelenburg position for its effect on VAE risk has a controversial history [19]; though, it has been shown to increase the pressure in the right atrium and balance the inward pressures of insufflation [13]. Hong et al. hypothesized that this mechanism is a contributory factor to the low rate of VAE in RARP with a steep Trendelenburg angle of 30 degrees, opposed to the typical 15 degrees used in gynecologic surgeries [42]. As the performance of these maneuvers during robotic laparoscopies is not possible due to the patient’s fixed position, it was excluded in the accompanying algorithm (Figure 3).

Air aspiration from the right atrium via pulmonary artery catheter is also a debated intervention. While it has been reported that 50% of entrained air can be aspirated, most studies find this treatment inconsistent in efficacy [46-48]. Further, inserting a catheter solely for the purpose of aspiration has been suggested as controversial due to its risk and low yield [17]. Current recommendations are strongest for its use in hemodynamically unstable patients who have a catheter in situ and are refractory to other management interventions [8,9,17]. This review found that 39% of case reports attempted to aspirate the right atrium for air; successful aspirations also note the beneficial diagnostic ability of this procedure [23,25,28-30,34,37,44,45]. Figure 3 compiles our results in a patient care algorithm. Article characteristics and descriptions are presented in Table 1.

**Table 1:** Literature review of VAE during laparoscopy VAE, Venous air embolism; IABP, intra-aortic balloon pump; LMA, laryngeal mask airway; ET, endotracheal; CPB, cardiopulmonary bypass; ASA, the American Society of Anesthesiologists; RALRP, robot-assisted laparoscopic radical prostatectomy; PFO, patent foramen ovale; RA, rheumatoid arthritis; CVC, central venous catheter; CPR, cardiopulmonary resuscitation; PEEP, positive end-expiratory pressure; SVC, superior vena cava; ECLS, Extracorporeal Life Support Program; NICU, newborn intensive care unit; PICU, pediatric intensive care unit; PDA, patent ductus arteriosus; BIS, bispectral index.

Lead Author (Year)	Country	Type of Article	Patient Characteristics	Case Management or Suggested Management
Kato et al. (2015) [6]	Japan	Case Report	A 76-year-old man underwent laparoscopic transurethral holmium laser enucleation of the prostate. Unknown if surgery was completed after VAE. He was discharged on POD 8 with no sequelae.	Stopped causal agent, 100% oxygen and pharmacologic agents given to maintain hemodynamics. IABP placed for support during ICU recovery.
Lee and Vazquez (2015) [7]	USA	Case Report	A 73-year-old male with no known cardiovascular history and benign prostatic hypertrophy underwent greenlight laser photoselective vaporization of the prostate for bladder outlet obstruction. Unclear if surgery was completed after VAE. The patient was discharged on POD 1 without sequelae.	100% O_2 _given, terminated insufflation, and LMA switched to ET tube for respiratory support.
Park et al. (2012) [9]	Korea	Review	N/A	Diagnosis with TEE, precordial Doppler, ETCO_2_ changes, and/or precordial or esophageal stethoscope ‘mill-wheel’ murmur. Stop insufflation, give 100% O_2_, discontinue nitrous oxide, hyperventilate. Give IV fluids, perform Durant maneuver, continuous assessment of vitals with pharmacologic agents to manage hemodynamics. Consider IABP or CPB.
Hong et al. (2010) [12]	Korea	Clinical Study: Retrospective	43 patients with ASA status of I or II scheduled for elective RALRP enrolled between June 2007 and November 2007. Patients with esophageal disease were excluded due to contraindications with TEE. Two patients enrolled were excluded due to failure of TEE insertion. No patients had PFO or cardiac shunt.	TEE detects subclinical VAE in 17.1% of laparoscopic radical prostatectomies.
de Jong et al. (2019) [14]	The Netherlands	Review	N/A	Terminate pneumoperitoneum, Durant maneuver, aspirate RA for air with CVC or PA catheter. CPR if needed.
Gainsburg et al. (2012) [15]	USA	Review	N/A	Close coordination between anesthesiologists and surgeons.
Orhurhu et al. (2020) [17]	USA	eBook	N/A	Immediate communication with the surgical team, terminate insufflation, Durant maneuver, hyperventilation with 100% O_2_, manage hemodynamics with pharmacologic agents. Attempt aspiration with CVC for high-risk scenarios.
Takechi et al. (2020) [18]	Japan	Case Report	A 59-year-old male underwent laparoscopic right hemihepatectomy for intrahepatic cholangiocarcinoma. Surgery was completed after VAE. The patient was discharged without further complications.	Ventilated at 100% O_2_, reduced pneumoperitoneum, and given pharmacologic agents for hemodynamic support. Trendelenburg position was performed and high PEEP was given.
De Cassai et al. (2019) [19]	Italy	Case Report	A 67-year-old female with a history of laparoscopic cholecystectomy underwent laparoscopic liver resection for hepatocarcinoma. Surgery was completed following VAE. The patient was transferred to the ICU and was discharged on POD 3 without further complications.	Pneumoperitoneum reduced, PEEP raised, and lesion clamped. Conversion not indicated. Aspiration performed.
Kale et al. (2019) [20]	USA	Case Report	A 44-year-old female with a history of severe supravalvular aortic stenosis with surgical repair at 24 years, atrial fibrillation without anticoagulation, and persistent left SVC underwent laparoscopic robotic rectopexy for recurrent rectal bleeding secondary to rectal prolapse. The surgery was converted to laparotomy and completed following VAE. Further course not mentioned.	Communicated with the surgical team, stopped insufflation, and pharmacologic agents are given to support hemodynamics. IV fluids are given, and an internal jugular catheter was inserted. TEE probe used to investigate. Aspiration of gas attempted. Durant maneuver performed. CPB considered.
DiChiacchio et al. (2018) [21]	USA	Case Report	A 5-day-old term male with a history of bladder outlet obstruction with anhydramnios requiring cystocentesis, multiple amniocenteses with amnioinfusion, and placement of both vesicoperitoneal and vesicoamniotic shunts underwent a laparoscopic peritoneal dialysis catheter placement for worsening renal function. The patient was placed on ECLS and transferred to the NICU. The patient expired after withdrawing ECLS, per the mother’s request.	Communicated with surgeons and stopped pneumoperitoneum. CPR performed. Aspiration of air embolism was attempted. Durant maneuver performed. Supported on ECMO.
Harnsberger et al. (2018) [22]	USA	Case Report	3 of 80 patients who underwent a transanal total mesorectal excision had a VAE from December 2014 to March 2018 at a single institution. No intraoperative or postoperative sequelae were reported including arrhythmia, myocardial infarction, stroke, or death. These surgeries were completed without conversion to open.	Stopped insufflation and given hemodynamic support with fluids and pharmacologic agents. Durant maneuver performed.
Basaran et al. (2016) [23]	Turkey	Case Report	An 8.5-kg 13-month-old female with Trisomy 21 and pectus carinatum underwent laparoscopic Morgagni Hernia Repair with a known PFO. The surgery was completed after VAE. The patient was discharged on POD 2 without sequelae.	Preventative management for patients with known PFO includes keeping intra-abdominal pressure under 12 mmHg, use of TEE, and avoiding nitrous oxide for suspected VAE.
Tognon et al. (2014) [24]	Italy	Case Report	A 12-year-old female, previously diagnosed and treated for Hodgkin Lymphoma, underwent a laparoscopic lymph node biopsy for suspected reoccurrence. Surgery was completed after VAE. The patient was transferred to the PICU and was discharged on POD 7 without sequelae.	Insufflation stopped. Durant maneuver performed. Aspirated via CVC. CPR was administered with 100% O_2_ and pharmacologic agents.
Olsen et al. (2013) [25]	Australia	Case Report	A 3.14-kg term 3-day-old male underwent a laparoscopic duodenoduodenostomy for duodenal atresia, with preoperative echocardiogram confirming small PDA with left to right flow and PFO. The procedure was abandoned, and the patient was transferred to NICU. On POD 7, the patient was extubated, and the surgery was attempted again. No sequelae at discharge on POD 15 and is meeting developmental milestones at 19 months of age.	Pneumoperitoneum stopped. chest compressions, pharmacologic agents, and 100% O_2 _given. The patient was placed in Trendelenburg.
Vora et al. (2013) [26]	India	Case Report	A 35-year-old female underwent a transperitoneal laparoscopic Boari’s ureteric reimplantation. Surgery was abandoned after VAE. The patient was transferred to ICU and regained consciousness 2 hours post-op. She was discharged without any neurological deficit.	Pneumoperitoneum released, placed in Trendelenburg, fluids and pharmacologic agents administered to maintain hemodynamics. Auscultation of precordial region was without ‘mill-wheel’ murmur. FiO_2 _increased to 100%. CPR given. Unsuccessful RA aspiration via CVC.
Shen et al. (2011) [27]	China	Case Report	A 72-year-old male without significant past medical history underwent elective laparoscopic right kidney resection. Surgery was completed after VAE, and the patient recovered without any sequelae.	Precordial auscultation revealed splashing ‘mill-wheel’ murmur. Aspirated RA via CVC. Pneumoperitoneum was terminated.
Smith et al. (2011) [28]	USA	Case Report	A 34-year-old female underwent suction D&C followed by a laparoscopic procedure to examine her uterus. Surgery was completed after VAE; the patient was discharged home without any long-term adverse events.	100% oxygen given, stopped insufflation, aggressive fluid resuscitation, and pharmacologic agents given to manage hemodynamics. Durant maneuver performed. The laparoscopic procedure was aborted.
Sandadi et al. (2010) [29]	USA	Review	N/A	Diagnose, stop insufflation, alert surgical staff, manage hemodynamics with pharmacologic agents, give 100% O_2_, and place in Trendelenburg position. If stable, discuss continuing surgery or converting to open procedure. If still unstable, call the cardiac arrest team, give CPR, and insert the right atrial catheter for gas aspiration.
Brull et al. (2017) [30]	USA	Review	N/A	Early action, terminate the source of air, stop communication between atmosphere and vessel, give high flow 100% O_2_. Perform aspiration via CVC only if already in place, low yield if the sole purpose for placement is aspiration. Perform Durant maneuver, CPR, and additional resuscitation therapy including the use of pharmacologic agents and ECMO.
Mills et al. (2011) [31]	UK	Review	N/A	Management includes careful monitoring of ETCO_2_. If VAE is suspected, infuse fluids rapidly, give 100% O_2_, and inform the surgeon who should prevent further air entering. Aspirate if the line is in situ. Consider using TEE and Doppler techniques to monitor.
Seong et al. (2010) [35]	Korea	Case Report	A 65-year-old male with prostate cancer underwent a laparoscopic prostatectomy. Surgery completed following VAE. Transferred to the ICU and discharged on POD 10 without any related complications.	Ventilated with 100% O_2_, stopped insufflation, IV fluids, and pharmacologic agents given, placed in Durant’s position, aspiration via CVC. TEE and CXR are used for evaluation. Converted to open surgery and recovered in the ICU.
Sollazzi et al. (2011) [36]	Italy	Case Report	A 43-year-old male, with mild hypertension and thyroiditis, underwent an elective retroperitoneoscopic right adrenalectomy for an adrenal ‘incidentaloma’. Surgery completed after VAE. Post-op patient was transferred to ICU. With an uneventful surgical course, he was discharged home POD 7.	Durant maneuver performed. 100% O_2_, pharmacologic agents, and IV fluids are given. TEE used.
Burcharth et al. (2012) [37]	Denmark	Case Report	A 50-year-old female without notable medical history underwent laparoscopic cholecystectomy and liver cyst fenestration. VAE occurred immediately after surgery was completed. The patient was transferred to another hospital for post-op care and was discharged on POD 14 without any sequelae.	Precordial Doppler ultrasound confirmed VAE. Durant maneuver was performed, 100% O_2_ given, CVC line placed, and air was aspirated.
Kawahara et al. (2017) [38]	Japan	Case Report	A 60-year-old male with hypertension and post-laparoscopic cholecystectomy underwent laparoscopic liver resection for hepatocellular carcinoma. Paradoxical VAE occurred with the absence of right-to-left systemic shunt. Surgery was completed after conversion to open laparotomy. Post-operatively, the patient was transferred to the ICU, went into a coma, and suffered quadriplegia. The patient was discharged to rehabilitation with severe neurologic sequelae remaining 6 months post-op.	TEE is used to show air in left atrium and left ventricle. BIS monitoring was introduced to evaluate brain activity after VAE was suspected to develop into a paradoxical CO_2_ embolism. The venous opening was closed, and surgery was converted to laparotomy.
Lee et al. (2019) [39]	Singapore	Case Report	A 71-year-old male with a history of DM, HTN, chronic renal impairment, dyslipidemia underwent elective laparoscopic liver resection for Childs A liver cirrhosis from hepatitis B complicated by hepatocellular carcinoma. Surgery was resumed after VAE with two more episodes of embolism. After completion, the patient recovered in the ICU, complicated by pneumonia. He was discharged 2 weeks post-op without other sequelae.	Communicated with the surgical team, pneumoperitoneum terminated, and placed on 100% O_2_ with pharmacologic agents given to support hemodynamics. VAE confirmed by aspiration of RA with CVC. Precordium auscultation found ‘mill-wheel’ murmur.
Abraham et al. (2018) [40]	India	Case Report	A 23-year-old female with previous endoscopic trans-sphenoidal radical excision for Cushing’s syndrome underwent bilateral adrenalectomy via retroperitoneoscopy. Surgery was successfully completed after VAE. She was transferred to the ICU and was discharged without related sequelae on POD 4.	Communicated with surgeon, FiO_2_ increased to 100%, and Durant maneuver was performed. Aggressively treated with fluids and pharmacologic agents. Aspiration via CVC was successful. Surgery completed.
Russell et al. (2018) [41]	USA	Reply to Case Report	N/A	Stop insufflation, perform Durant maneuver, give 100% O_2 _with aggressive volume expansion and pharmacologic agents for hemodynamic support. CPR as needed and if severe, consider aspiration. Discuss the decision to continue by surgeon and anesthesiologist.
Taylor et al. (2010) [42]	USA	Case Report	A 3.6-kg term 12-day-old female was admitted for volume resuscitation and evaluated for suspected pyloric stenosis. Laparoscopic pyloromyotomy was performed. VAE occurred with neurologic manifestations due to a confirmed PFO. Surgery still completed. MRI showed watershed infarcts post-op. Normal neurologic exam at the time of discharge on POD 9. More than two years later, the patient is meeting a developmental milestone.	CPR was performed, and pharmacologic agents are given for hemodynamic control. Other etiology is ruled out.
Galipienzo et al. (2013) [43]	Spain	Case Report	A 58-year-old male underwent laparoscopic left hemicolectomy for tumor in the colon. The surgical procedure was suspended after VAE. Case conclusions were not reported.	Fluids given. Rule out other etiologies. Pharmacologic agents are given to maintain hemodynamics. Surgical procedure suspended. CT ruled out pulmonary embolism and DVT. VAE was diagnosed by exclusion.
Yu and Fang (2014) [44]	China	Case Report	A 52-year-old female underwent laparoscopic nephrectomy. Surgery was converted to open procedure and was completed after VAE. The patient was transferred to the ICU with mechanical ventilation support for pulmonary edema. The patient was extubated on POD 3 and was discharged without any sequelae on POD 11.	Insufflation discontinued. Trendelenburg maneuver performed followed by CPR with pharmacologic agents given to maintain hemodynamics. TEE confirmed VAE.
Hong et al. (2010) [47]	Korea	Clinical Study: Retrospective Study	Patients with ASA physical status I or II undergoing RALRP (n = 26) or RRP (n = 26) were enrolled from March 2007 to November 2007. One patient was excluded due to TEE insertion failure.	TEE can be used to detect subclinical VAE during RALRP at 38% and RRP at 80%.
Yamashita and Nishida (2016) [49]	Japan	Review	N/A	Close coordination among the surgeons, anesthesiologists, and other medical staff.
Shiraishi et al. (2018) [50]	Japan	Review	N/A	The key is prevention and early identification of CO_2_ embolism with active and effective symptomatic treatment including reducing pneumoperitoneum and using TEE as a sensitive way to monitor VAE.
Eser et al. (2016) [51]	Turkey	Review	N/A	Release pneumoperitoneum and stop insufflation. Perform Durant maneuver. Aggressive volume expansion or aspiration by insertion of CVC may be attempted.
Lam et al. (2010) [52]	Australia	Review	N/A	Release pneumoperitoneum. Exclude alternative etiologies including a cardiac event, anaphylaxis, or intra-abdominal hemorrhage.

Based on our case and a literature review, we developed a management algorithm for use when clinical VAE presents during robotic laparoscopies. This algorithm presents key decision points driving appropriate and validated monitoring and management addressing exposure risks of venous systems and assessing improvement versus stability. Ultimately, this algorithm is designed to work in synergy with a working knowledge of VAE physiology, diligent monitoring, and open communication among the perioperative team.

The management of carbon dioxide embolism has been previously described [8], amended with a laparoscopy focus [9], and revised in 2017 [17]. In comparing the case report management practices to what was covered in these reviews, most were consistent with management guidelines; however, there were some expected variations. Consistent practices among case reports included the use of ETCO_2_ and SpO_2_ as primary indicators of VAE, termination of insufflation, use of 100% oxygen, and maintenance of hemodynamics with intravenous pharmacologic agents and fluids. In contrast, the application of more sensitive VAE detection technology like precordial Dopplers was under-utilized, although actual appropriateness is difficult to evaluate. The intervention of air aspiration was used in about half of the case reports. This inconsistency could be due to the perceived risk versus benefit involved in inserting the central catheter or that the embolic event resolved before the intervention was able to be applied.

Most cases and reviews do not comment on the importance of adequate communication as part of a treatment algorithm. While it is conceivable that proper communication is so commonplace that it is not considered a necessary step to mention, it is nonetheless harmless and imperative to optimal patient safety, cannot be understated, and is thus included in our management algorithm. In the same vein, the decision to continue or abort surgery also lacks commentary in the literature. It seems intuitive that these decisions are considered and risks stratified by the anesthetic and surgical teams as per patient stability. A specific approach to preventative mechanisms to avoid a second VAE during these cases is also not generally addressed in the articles reviewed.

Finally, post-procedural management is an important component to complete any patient care algorithm. While the detection of neurological sequelae is an important aspect to continued post-anesthetic care, some highlight the importance of monitoring right heart failure post-operatively [9] with others underscoring the importance of mitigating the VAE effect on right ventricular afterload as a precursor to causing heart failure [8].

Future research would benefit from validating our presented algorithm in managing VAE during RARP. Specific areas that merit further illumination include decision support to guide continuing or aborting surgery and VAE-specific post-procedure monitoring and support. Barriers to using supplemental monitoring, like precordial Doppler ultrasound, should be further evaluated to best understand their lack of widespread use.

## Conclusions

In conclusion, we report successful management of VAE during robotic-assisted laparoscopic prostatectomy. Upon debriefing, we identified the lack of an adequate management algorithm, given the constraint from using the robot and accompanying patient position. VAE identification is challenging, and severe sequelae can occur suddenly and rapidly. Reviewing the literature to date, we provide an updated patient care algorithm in an effort to promote a standardize approach to VAE management during robotic-assisted laparoscopic procedures.
